# Factors Influencing the Consumption of Seaweed amongst Young Adults

**DOI:** 10.3390/foods11193052

**Published:** 2022-10-01

**Authors:** Mikaela Young, Nicholas Paul, Dawn Birch, Libby Swanepoel

**Affiliations:** 1 School of Health and Behavioural Sciences, University of the Sunshine Coast, Sippy Downs, QLD 4556, Australia; 2 School of Science, Technology and Engineering, University of the Sunshine Coast, Sippy Downs, QLD 4556, Australia; 3 School of Business and Creative Industries, University of the Sunshine Coast, Sippy Downs, QLD 4556, Australia; 4 Australian Centre for Pacific Islands Research, University of the Sunshine Coast, Sippy Downs, QLD 4556, Australia

**Keywords:** consumer behaviour, motivator, barrier, packaging, alginate, sustainability, marketing, diet, snack

## Abstract

Seaweed has been traditionally consumed in Asian and Pacific cultures, yet aside from sushi, is still not commonly eaten in Western societies. Edible seaweeds offer distinct nutritional benefits to terrestrial crops, particularly with respect to mineral and fibre content. Understanding the motivations that drive young Australians to eat seaweed is necessary for food product development and consumer marketing strategies, as well as informing future sustainable production through seaweed aquaculture and wild-harvest practices. An observational cross-sectional online survey with *n* = 1403 young (19–30 years) Australian seaweed consumers was conducted. The 19-item survey included closed-ended, open-ended, and Likert scale responses. Most respondents were female (89.0%), with tertiary level education or above (57.7%). Seaweed was eaten mostly as a snack (87.7%) and in home-prepared meals (30.7%). The key advantages to consumption were flavour (89.1%), nutrient content (49.1%), and health benefits (44.6%), whilst the key barriers were poor accessibility (59.5%), unaffordable pricing (46.5%), and undesirable packaging (19.0%). The consumers reported wanting more promotion to improve their knowledge about seaweed, in addition to environmentally sustainable packaging and sourcing. Pathways to overcome barriers and encourage greater seaweed consumption are discussed. Most critically, improving the promotion and environmental sustainability of seaweed products will improve intake amongst current and future consumers.

## 1. Introduction

Edible seaweeds have been traditionally consumed across coastal communities, including regions within China, Japan, the Republic of Korea, and across the Pacific [1,2,3,4]. The consumption of seaweed remains a growing trend in Western countries [5], often associated with the migration of consumers who eat traditional seaweed dishes to Western countries [1]. Seaweeds are classified into red, brown, and green taxonomic groups with more than 10,000 species presently identified [6,7]. Some well-known dishes traditionally made with seaweed include sushi, salads, pickled seaweed with condiments such as vinegar or relish, and soups [8]. Popular seaweed-containing foods amongst Australian consumers include sushi, seaweed-flavoured crackers, seaweed soup, and seaweed-flavoured snacks [9].

Seaweed offers a range of micronutrients to the diet, with the nutrient content varying based on species, environmental characteristics, water temperature, and the nutrient content of the water [10,11,12]. Seaweeds can provide a good source of dietary fibre, calcium, magnesium, iron, folate, and iodine [5]. In regions where seaweed is traditionally eaten, seaweed contributes to the recommended daily vegetable intake [13,14,15]. Given only 7.5% of adult Australians consume the recommended serving of vegetables [16], there is potential for the addition of seaweed to contribute towards meeting vegetable recommendations and, hence, lowering the risk of diet-related noncommunicable diseases [17]. Additionally, the multi-faceted contribution that the food sector makes to environmental degradation is acknowledged within Australia [18]. Seaweed farming offers more positive environmental impacts, as cultivation places less demand on fresh water and arable land compared to terrestrial-grown crops [19]. The introduction of seaweed into the diet of more Australian consumers therefore provides the potential to complement terrestrial crops with an ocean-based, sustainable source of nutrients.

As seaweed is a non-traditional food for most Australian consumers, it is likely considered a novel food by many, similar to other Western countries [20,21]. There are several factors that influence whether a consumer accepts or rejects a food item, including the body of information about the food, the external social context, and the physical experience during and after consumption [22]. Additionally, the motives contributing to food choice differ across cultural groups and populations [23]. The characteristics of the food that contribute to consumer acceptance include the degree of food involvement, sensory appeal, health concern, mood, ethical concern, convenience, familiarity, and neophobia [24]. Food neophobia is a fear of trying unfamiliar foods, and the greater one’s degree of neophobia, the greater the reluctance to taste an unfamiliar food due to perceived unpalatability [25]. Hence, there are numerous factors to overcome when addressing consumer perception of seaweed, especially in countries or within demographics that are not traditionally consumers of seaweed.

Young consumers have been identified as the demographic most likely to eat seaweed in Western cultures [9,20]. More specifically, Australian seaweed consumers are likely to be aged 18–35 years, female, with a high level of education and high household income [9,20]. While we have some understanding of the profile of seaweed consumers in Australia, there is no study investigating the drivers of consumption specifically within this group of young adults. To encourage young Australian consumers to eat seaweed, we must first gain insight into what seaweed-eating occasions look like. Understanding current motivators and consumption habits can inform the development of future public health nutrition interventions and also guide food industry developments and consumer marketing. Due to dissensus amongst the existing literature regarding age grouping, a subset of young adults aged 19–30 years has been defined for the purpose of this research, mirrored from the groupings previously utilised by the Australian Bureau of Statistics (ABS) [26]. This study is a first-time exploratory study that seeks to understand consumer preference and motivators to seaweed consumption within young Australians. This is particularly relevant, as Australians aged 20–30 years made up 14.1% of the nation’s population in 2020 [27]. Besides being the largest proportion of seaweed consumers to date, young adults also possess the ability to influence the normalisation of seaweed intake, thereby reshaping healthier consumption behaviours for future generations.

## 2. Materials and Methods

### 2.1. Participants

An observational cross-sectional survey was undertaken amongst young Australian consumers (19–30 years) who actively eat seaweed. This was defined as self-reported consumption of seaweed on at least one occasion within the past twelve months. Recruitment occurred via convenience sampling [28] from June to August 2021 across the social networking sites Facebook, Instagram, and Twitter. Purposive sampling techniques were also employed using targeted social media advertisements. Purposive sampling is a common technique in qualitative research to identify and select information-rich cases related to the phenomenon of interest [29]. Purposive sampling through online recruitment via social networking sites is a useful approach to engage large samples of consumers who are otherwise difficult to access [30]. The participants were excluded if they were outside of the target age range (<19 or >30 years), resided outside of Australia, and/or were not able to complete the survey in English or on their own behalf. A sequential survey was used to obtain identifiable variables for participants who wanted to enter the prize draw, which was the chance to win one of three three-month subscriptions to a delivered gift box containing newly released health food products.

### 2.2. Data Collection

The 19-item survey was designed to assess consumer beliefs and consumption habits regarding seaweed (Table S1). The survey was developed and distributed using Qualtrics XM software. Due to the lack of validated tools to assess the factors influencing seaweed consumption, the researchers developed a prototype survey that included an adapted version of the tool used by Hicks et al. to assess consumer beliefs and knowledge regarding seafood consumption [31], and the validated Food Neophobia Scale (FNS) [25]. The Likert scales used to assess consumption patterns were informed by the Likert scales used in the Australian Eating Survey (AES) [32] and Dietary Questionnaire for Epidemiological Studies (DQES) v3.2 [33]. For ease of interpretation, the results discuss amended versions of the Likert scales presented in Questions 12 and 18.

The survey was piloted on two occasions, initially amongst young adults in February 2021 (*n* = 17), and then via convenience sampling with university students in June 2021 (*n* = 6). The feedback was used to revise the survey content for improved readability and structure flow. The survey consisted of six sections: (i) inclusion criteria, (ii) demographics, (iii) beliefs, (iv) sources of seaweed-related information, (v) consumption patterns, and (vi) FNS. The tool used a combination of close-ended questions, open-ended questions, and various pointed-Likert scales. There was no time restriction enforced on the survey nor the requirement for participants to respond to all questions to progress through the questionnaire.

### 2.3. Data Analysis

The quantitative data were analysed using the Statistical Package for the Social Sciences (SPSS) v27.0 software (Chicago, IL, USA) and were reported on using descriptive statistics, including frequencies and percentages. In the instance where there was less than a 100% response rate, the results are presented as an adjusted percentage with reference in the table or figure legend. The analysis of the FNS required that reverse-score items be first reversed and then a total score determined for each respondent. The scores were classified by the degree of neophobia, determined using the sample mean and standard deviation [34,35,36]. A Cronbach’s alpha value was calculated to evaluate the internal consistency of the scale [37]. Respondent postcodes were classified as either metropolitan, rural, or metropolitan/rural, based on the Regional Postcode Delivery Classifications of the Department of Agriculture, Water, and the Environment [38].

Tests were conducted using Pearson’s chi-square to identify statistical significance between the datasets (*p* ≤ 0.05). Due to the minimum expected cell count necessary to perform a valid test, many of the values for each variable were collapsed or omitted as described in the relevant Section of the Results. One example of this was collapsing the 6-point Likert scale in Question 12 rating importance down to binary important (all three important) and non-important (all three non-important). The multidimensional scaling method was also used to investigate the associations between the binary variables. Additionally, Question 18 asked participants to report on the frequency of consumption of a maximum of two fast food/takeout options. For ease of interpretation, the data captured relating to the second fast food/takeout option revealed no new values and, resultantly, are not presented in the results.

The qualitative data were analysed using content analysis [39]. The data were collated using Microsoft Excel 2018 and then read and re-read for familiarisation before allocating initial codes. Triangulation occurred by three researchers for a subset of the responses, independently assigning codes with descriptions and rationale. Questions 8–11 invited participants to provide multiple responses to each question (Q8: Please list three of the greatest advantages of eating seaweed (what are the three best things about eating seaweed); Q9: Please list three of the greatest disadvantages of eating seaweed (what are the three worst things about eating seaweed); Q10: Please list three factors that would enable (make it easier) for you to eat seaweed; Q11: Please list three factors that make it difficult (harder) for you to eat seaweed). In the instance where multiple responses from the same participant were coded as the same theme, only one response was counted. Responses were coded as multiple themes as necessary. Related codes were then collapsed into themes by two of the researchers in a collaborative method to ensure intercoder reliability, whereby each researcher coded the data independently before coming together and agreeing upon a final set of themes [30]. The qualitative data were then coded into quantified responses, transferred to SPSS for statistical analysis, and reported as frequencies and percentages. Selected quotes to represent each theme were chosen based on the quality and distribution across participants and subthemes.

### 2.4. Ethical Approval

This project was granted ethics approval from the Human Research Ethics Committee at the University of the Sunshine Coast, Queensland, Australia (S201510). The research was carried out through a self-administered online survey, where participants were required to provide consent in response to the Research Project Information Sheet prior to the commencement of the survey.

## 3. Results

### 3.1. Participant Characteristics

Of the 2007 participants, a total of *n* = 1403 respondents were included in the final analysis, having met the inclusion criteria, and completed ≥75% of the survey. The sample characteristics are presented in Table 1. Both age categories were represented by approximately half of all participants. Most respondents were female (*n* = 1248, 89.0%) and lived in a metropolitan area (*n* = 1095, 78.0%). Over half of all respondents had completed tertiary level education or above (*n* = 810, 57.7%), and half of all respondents had an annual household income above $60,000 (*n* = 717, 51.1%). Most of the participants resided in New South Wales (*n* = 442, 31.5%), Victoria (*n* = 360, 25.7%), and Queensland (*n* = 334, 23.8%).

### 3.2. Food Neophobia

The mean and standard deviation of the degree of neophobia amongst the sample was determined (25.19 ± 8.84) and then used to classify most participants (*n* = 1380, 98.3%) by degree of neophobia. Approximately two-thirds of all respondents (*n* = 938, 66.9%) scored as having an average food neophobia level, with 15.1% (*n* = 212) scored as having a low degree of neophobia, and 16.4% (*n* = 230) with a high degree of neophobia. The Cronbach’s alpha for the FNS was α = 0.868, indicating good internal consistency.

### 3.3. Influences on Decision to Purchase Seaweed

All participants (*n* = 1403) rated the importance of a variety of factors on their decision to purchase seaweed (Table 2). For the purpose of analysis, importance was classified as a cumulative result of ‘moderately important’, ‘very important’, and ‘extremely important’. The three most-reported influences were taste (*n* = 1328, 94.7%), cost (*n* = 1191, 84.9%), and ease of preparation (*n* = 1082, 77.1%). Participants aged 25–30 years were more likely to report health as an influence on the decision to purchase seaweed (χ^2^ analysis, *p* = 0.017) than participants aged 19–24 years. Female participants reported the importance of health more frequently than participants of other genders (*p* = 0.018). Participants with a high school-level education or equivalent were more likely to report friend/family preference as an important factor than participants with a tertiary-level education, equivalent, or above (*p* = 0.029). Participants who earnt an annual household income less than $40,000 per year were more likely to report cost as an important influence compared to those earning more than $100,000 per year (*p* = 0.004). Meanwhile, participants who reported earning more than $100,000 per year were more likely to report ease of preparation as an important influence compared to their lower-earning counterparts (*p* = 0.027). Participants who lived in metropolitan areas were more likely to rate ease of preparation (*p* = 0.006) and freshness (*p* = 0.044) as important than participants living in rural areas. Participants with high food neophobia were more likely to rate ease of preparation as important than their low neophobia counterparts (*p* = 0.006).

### 3.4. Source of Seaweed-Related Information

**Figure 1:**
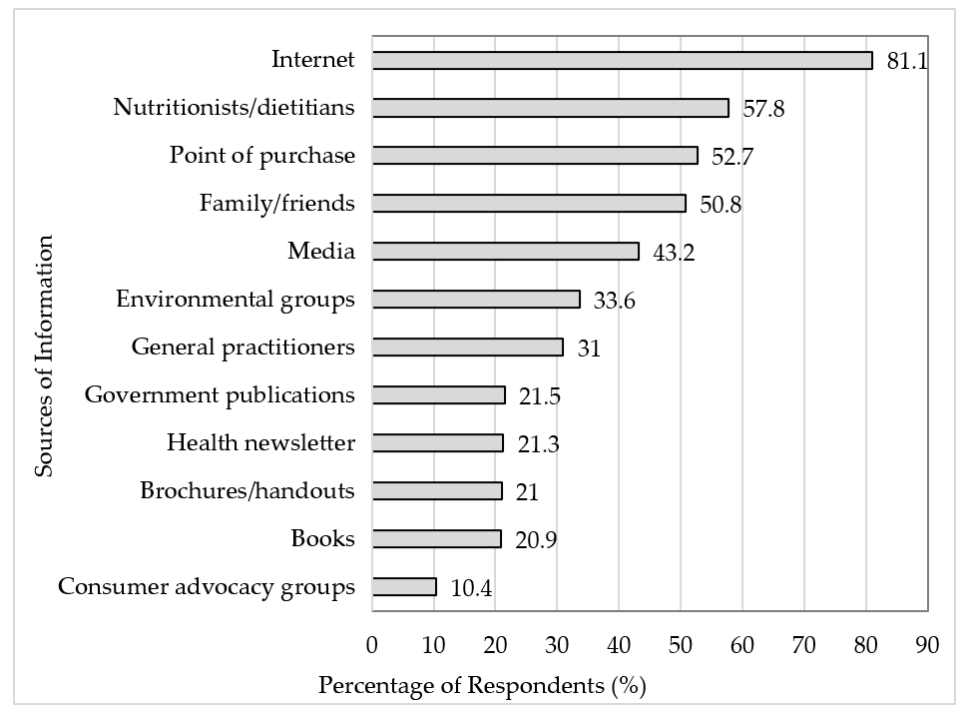
Current sources of seaweed-related information (*n* = 1403). The sources are presented in descending order from most to least reported. The data labels are representative of the percentage of respondents.

The entirety of the sample (*n* = 1403) reported utilising several sources to access seaweed-related information (Figure 1). The three most-reported sources were the internet (*n* = 1138, 81.1%), nutritionists/dietitians (*n* = 811, 57.8%), and the point of purchase (*n* = 739, 52.7%). All participants (*n* = 1403) then reported on their preferred source of seaweed-related information, with the three most preferred being the internet (*n* = 699, 49.8%), nutritionists/dietitians (*n* = 170, 12.1%), and the media (*n* = 126, 9.0%).

### 3.5. Familiar Forms of Seaweed

**Figure 2:**
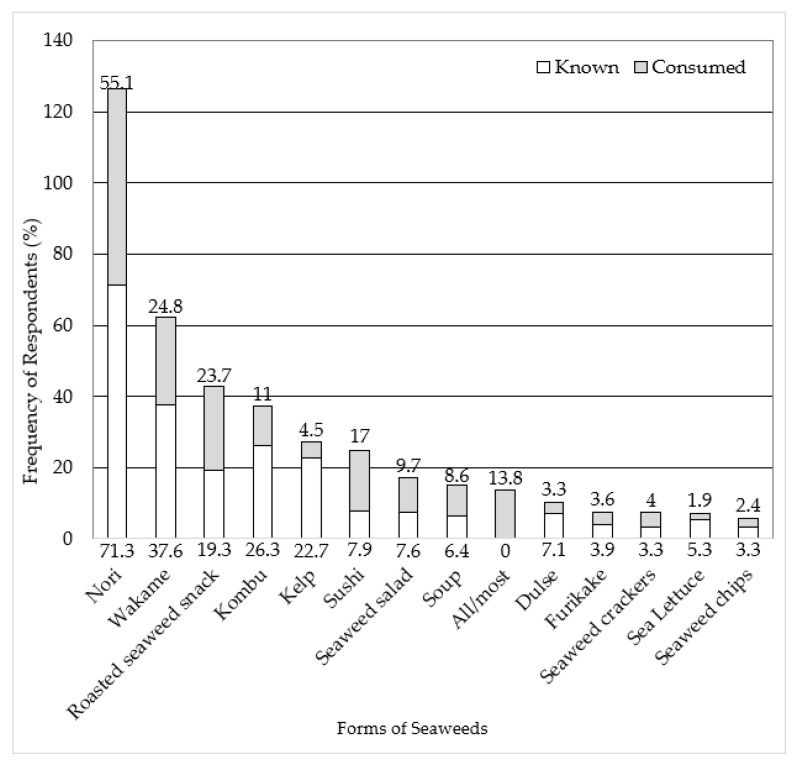
Forms of seaweed known and consumed, as reported by participants. The varieties are presented in descending order from most to least reported, cumulatively, of both known and consumed responses. The data labels are representative of the percentage of respondents.

Over half of the respondents reported on the forms of seaweeds (i.e., species, food item, dish) they were familiar with (*n* = 831, 59.2%). Familiarity was defined as either knowing of or having consumed the form of seaweed. Interestingly, this suggests that over one-third of all participants may not be aware of the form of seaweed they typically consume. Figure 2 shows the most-recalled form of seaweed known and consumed, defined by being reported by at least 5.0% of the respondents. The most-known forms of seaweeds included nori (*n* = 533, 71.3%), wakame (*n* = 281, 37.6%), and kelp (*n* = 170, 22.7%), with nori (*n* = 430, 55.1%), wakame (*n* = 194, 24.8%), and roasted seaweed snacks (*n* = 185, 23.7%) being the most consumed.

### 3.6. Eating Occasions

**Figure 3:**
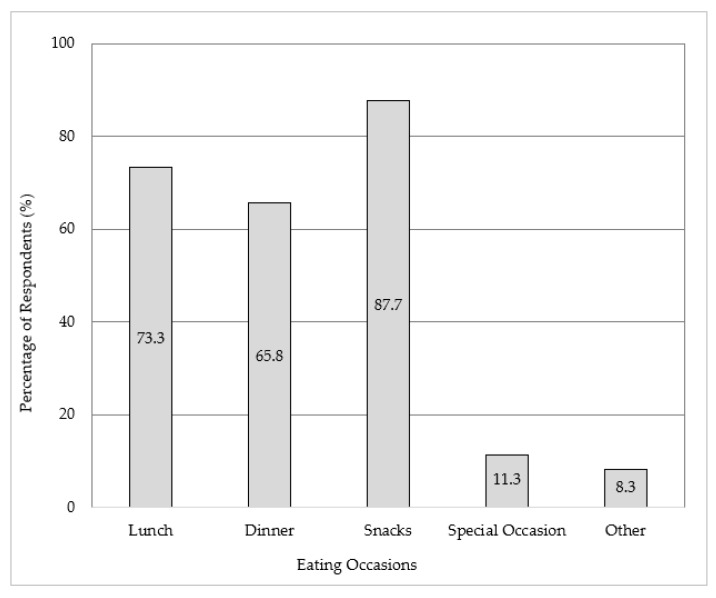
Popularity of seaweed consumption across eating occasions (*n* = 1402). The eating occasions are presented in the figure in the way that they appear in the survey question. The data labels are representative of the percentage of participants that responded to each eating occasion.

Almost the entire cohort reported on the eating occasions where seaweed was consumed (*n* = 1402, 99.9%) (Figure 3). The most common occasions were snacks (*n* = 1229, 87.7%), lunch (*n* = 1029, 73.4%), and dinner (*n* = 922, 65.8%). Additionally, 11.3% (*n* = 159) of respondents reported special occasions, and 8.3% (*n* = 116) reported ‘other’. The respondents were then asked to specify the eating occasion. The most commonly reported special occasions were dining at restaurants (*n* = 41, 31.5%), holidays and celebrations (*n* = 40, 30.8%), and sushi (*n* = 34, 26.2%), whilst ‘other’ occasions included breakfast (*n* = 32, 37.6%) and sushi (*n* = 19, 22.4%). Participants residing rurally were more likely to report consuming seaweed as a special occasion (χ^2^ analysis, *p* = 0.042) compared to metropolitan-residing participants. Participants with low neophobia were more likely to consume seaweed as a snack (*p* = 0.008) and at dinner (*p* < 0.001) compared to participants with high food neophobia.

### 3.7. Frequency of Consumption

The entire sample (*n* = 1403) reported on how frequently seaweed was consumed in different settings (Table 3). For the purpose of analysis, ‘frequent’ was defined as once fortnightly or more often. The three most common settings of consumption were home-prepared (*n* = 431, 30.7%), sushi bar/train (*n* = 359, 25.6%), and restaurant (*n* = 216, 15.4%). The fast food/takeout setting was the least common setting, as reported to be frequented by only 10.9% (*n* = 153) of participants. Some participants (*n* = 247, 17.6%) then reported on the ‘fast food/takeout’ setting that they consume seaweed in, with the most common responses being sushi (*n* = 126, 51.0%), Southeast Asian cuisine (*n* = 65, 26.3%), ramen (*n* = 26, 10.5%), soup (*n* = 20, 8.1%), and poke/rice bowl (*n* = 15, 6.1%). Participants aged 19–24 years were more likely to report consuming seaweed at a restaurant (*p* = 0.004), sushi bar/train (*p* = 0.001), and as fast food/takeout (*p* = 0.043) than participants aged 25–30 years. Participants with high school-level education or equivalent education level were more frequently consuming seaweed at a restaurant (*p* < 0.001) and at a sushi bar/train (*p* = 0.004) than their higher-level education counterparts. Queensland residents were more likely to report that they consumed seaweed at sushi bars/trains (*p* < 0.001) and restaurants (*p* = 0.014) than Victorian and New South Wales residents. Participants in metropolitan locations were more likely to report consuming seaweed at a restaurant (*p* = 0.005) and at a sushi bar/train (*p* = 0.023) than participants in rural locations. Participants with low neophobia consumed seaweed at a restaurant (*p* < 0.001) more frequently than their highly neophobic counterparts.

### 3.8. Associations between Demographic Characteristics, Beliefs, and Consumption Patterns

The multidimensional scaling method was used to analyse the associations between the binary data including demographic characteristics, beliefs, and consumption patterns. The respondents who snacked on seaweed were mostly from metropolitan areas, and typically identified taste, friend/family preference, cost, and ease of preparation as the most important influences on their decision-making regarding seaweed. Interestingly, they were less likely to report eating seaweed in sushi bars/trains. Furthermore, the respondents with a high degree of neophobia were younger, with a lower education level and lower income. Additionally, they rarely consumed seaweed in restaurants or for dinner.

### 3.9. Motivators and Barriers of Consumption

The participants were asked to list the perceived advantages (*n* = 1401, 97.3%), disadvantages (*n* = 1336, 95.2%), enablers (*n* = 1353, 96.4%), and barriers (*n* = 1246, 88.8%) associated with seaweed consumption. A qualitative content analysis of these open-ended responses generated several common themes. Table 4 presents the themes that correlate to ≥5.0% of respondents, with selected quotes to represent each theme. The three most commonly reported advantages were flavour (*n* = 1248, 89.1%), nutrient content (*n* = 688, 49.1%), and health benefits (*n* = 625, 44.6%), while some of the least reported included complementing the dish (*n* = 33, 2.4%) and trendiness (*n* = 29, 2.1%). The three most-reported disadvantages were undesirable side-effects (*n* = 508, 38.0%), unaffordable price point (*n* = 429, 32.1%), and lack of accessibility (*n* = 399, 29.9%), while some of the least reported included easiness to overconsume (*n* = 46, 3.4%) and unenjoyable by itself (*n* = 14, 1.0%). The three most commonly reported enablers were greater accessibility (*n* = 972, 71.8%), affordable price point (*n* = 621, 45.9%), and desirable packaging (*n* = 391, 28.9%), while some of the least reported included greater convenience (*n* = 45, 3.3%) and desirable nutrient content (*n* = 31, 2.3%). The most frequently reported barriers were lack of accessibility (*n* = 741, 59.5%), unaffordable price point (*n* = 579, 46.5%), and undesirable packaging (*n* = 237, 19.0%), while some of the least reported included lack of versatility (*n* = 53, 4.3%) and unsatiating (*n* = 25, 2.0%). Interestingly, a common theme that emerged as both a disadvantage (*n* = 192) and barrier (*n* = 141) to consumption was the excessive plastic packaging used in retail seaweed products.

## 4. Discussion

The findings from our study indicate that young Australian seaweed respondents are predominately female, well-educated, with a high household income and a low degree of neophobia. The most commonly reported advantages of seaweed consumption were flavour, nutrient content, and health benefits, whilst the most frequently reported disadvantages were undesirable side-effects, unaffordable price point, and lack of accessibility. Consumer beliefs and perceptions regarding seaweed have been explored previously [9,20,40,41,42,43]. However, to the best of our knowledge, this is the first study focusing on consumption amongst young adults, arguably the most important demographic given their influence as the largest proportion of seaweed consumers.

Seaweed is an important source of micronutrients to half of the young respondents in our sample. Many indicated that the main driver to consumption is the iodine content. The most known and consumed form of seaweed, nori, when consumed in small quantities, can provide enough iodine to meet or exceed the recommended dietary intake (RDI) of 150 µg/day [44,45]. This also applies to roasted seaweed snacks, which were the third most-consumed form and primarily consist of nori, as one snack-sized packet can provide enough iodine to meet the RDI [46]. Iodine is necessary for many aspects of regular growth and metabolism, namely in foetal development [45], yet the current median intake in Australia is 124 µg/day [47]. Interestingly, the iodine content of nori snacks is not commonly reported on the nutrition information panel of products available in Australia, although protein, fat, carbohydrates, and sodium are. In addition to potentially fulfilling one’s iodine requirements, seaweed also contributes to daily vegetable intake, which we know is not being achieved by the vast majority of Australians [16]. So, how do we encourage greater seaweed consumption to take advantage of the benefits seaweed offers, including mitigating the risk of micronutrient deficiencies, and contributing to daily vegetable servings? Most respondents were driven to consume seaweed based on the flavour profile and pleasurable snack options. Vegetable-based snacks are emerging into the competitive snacking landscape [48], providing a timely opportunity to encourage consumption via seaweed-based snacks. However, consideration is necessary to preserve the nutritional composition of seaweed when being processed into snack foods to maintain the health benefits that it is praised for. Herein also lies the opportunity for the nutritional composition of seaweed products to feature on the packaging, to mitigate respondent concern for the under- and overconsumption of micronutrients as a barrier to intake.

Young Australian respondents considered the lack of product diversity to be a key barrier to seaweed consumption. However, more than one-third of the respondents were unable to identify a single species of seaweed (i.e., specific types, taxonomic or common names of seaweed). The targeted promotion of existing seaweed-based food items is warranted to boost awareness and encourage diverse seaweed consumption. Young respondents want to access seaweed-related information via the internet, with promotional content offering valuable material such as recipes and education. It is instructive to evaluate the promotional strategies of other non-traditional foods currently assessed for food acceptance amongst Western consumers, such as the consumption of insects. Batat and Peter advocate that insect consumption should be promoted through marketing and advertising strategies, taking into consideration consumer self-esteem, food literacy, and the importance of value-based messaging regarding environment and sustainability [49]. These strategies are consistent amongst the literature discussing greater seaweed consumption amongst Western consumers [20,50]. There may also be merit in encouraging seaweed consumption amongst current consumers, with the intent that this will have positive flow on effects by improving the acceptance of seaweed amongst non-seaweed consumers [21]. Hence, an upstream approach to encourage consumption amongst the greater community could begin with targeted promotional material directed towards current consumers.

The young respondents reported that the current packaging is a barrier to consumption, with many attributing this to excessive single-use plastic. We found that seaweed consumption was intrinsically related to the notion of environmental sustainability, yet young respondents identified the irony in the unsustainable packaging typically encasing seaweed products. Emerging innovations have been exploring seaweed as a material to produce sustainable plastic. Two notable brands are Evoware and Notpla, who produce biodegradable and edible seaweed-derived packaging as alternatives to traditional plastic [51,52]. These innovations could be fast-tracked when aligned to national strategy, for example, Australia’s 2025 National Packaging Targets [53]. Environmentally conscious respondents also raised concern for the lack of local and organic varieties available. Local regions across Australia boast the ideal conditions for seaweed cultivation [54], with some native species offering similar palatability compared to widely accepted seaweed varieties [55]. Herein lies the potential for local seaweed supply chains, offering native species encased in biodegradable packaging. In addition to significantly reducing food miles and landfill contribution, authentically sustainable seaweed options will encourage consumers to align their personal values with consumption.

The respondents of this study are predominantly female, well-educated, of high income, and residing in metropolitan areas, which is consistent with the international literature [9,20,41,42]. Similarly, the association between a lower degree of neophobia and greater acceptance of seaweed is a common thread in other seaweed studies [9,21,41]. The young Australian respondents were primarily motivated by flavour, nutrient content, and health benefits [9,43]. The young Australians reported key enablers such as greater accessibility, affordable price points, and desirable packaging methods, which were generally consistent with the findings of Buehrlen et al. [40]. The key barriers identified in the present study differ from previous research, and we accentuate here the reported lack of accessibility, unaffordable price point, and excessive packaging. Previous literature identified alternative barriers such as negative health effects [41], location of origin [41], high iodine content [40], lack of knowledge [9], unpalatable flavour [43], and hygiene concerns regarding sourcing [41,43]. This difference may be explained by the differing survey tools used across the research studies. While two of the previous studies utilised close-ended surveys [41,42,43], and the other a mall-intercept-style survey (also closed-ended) [40], our findings were captured through open-ended questioning relying on qualitative analysis. This method allowed respondents to identify key barriers in their own experience without being influenced or prompted to select from predefined options.

The recruitment process in our study included a paid Facebook advertisement, allowing researchers to consciously recruit the intended audience through specifying consumer demographics that aligned with the inclusion criteria [56]. These demographics are consistent with the existing literature [9,20,41], and the geographical location of consumers is proportional to the Australian states and territories with the greatest populace [57]. Furthermore, the sample mean of the degree of neophobia (25.19 ± 8.84) was lower than the median score of the FNS tool [41], suggesting that the sample may have an overall lower degree of neophobia than a generalisable sample group. This matches our intent to recruit participants who consume a non-traditional food within the Australian context, which reinforces the construct that the intended consumers were captured in this sample. When considering the large proportion of female respondents, it is also important to consider that the majority of consumers exposed to the recruitment material identified as female, and that females are more likely to complete online surveys compared to their male counterparts [58].

There are potential biases associated with any recruitment approach [59], and so it is acknowledged that the sample here is not representative of the greater population. The current study is not without other limitations. Firstly, as recruitment occurred via targeted convenience sampling, it is possible that this sample is over-represented by females, hence, future research could investigate the influence on consumption amongst genders, noting that non-binary respondents were more represented than males in this study. There is potential that Facebook advertisements may be useful to further recruit non-binary consumers, given their higher level of representation in this study. The results may indicate a greater interest in healthy food consumption amongst females; however, more research would be needed to explore this. Participant nationality was not included in the survey tool, which meant we could not explore the relationship between neophobia and seaweed consumption and the influence of nationality and culture over seaweed perception. Hence, future research could capture ethnicity amongst other demographic characteristics to capture a holistic understanding of ethnic influential factors on seaweed consumption.

## 5. Conclusions

There is now a greater understanding of what seaweed consumption amongst young Australians looks like and what drives this consumption. Young consumers are motivated by flavour, nutrient content, and health benefits, yet are deterred by lack of accessibility, unaffordable pricing, and lack of diversity of options. Young Australians are asking for the greater promotion and marketing of seaweed products, and for an alternative to the excessive single-use plastic packaging. These findings can be harnessed to inform consumer marketing strategies and food product development, and to provide initial insight into the target market segmentation of seaweed. Future research should involve qualitative research to more explicitly understand how consumer perspectives and experiences influence intake, guided by the motivators and barriers identified in this study. Additionally, this study gives merit to the notion of a modelling study that measures the impact of regular daily seaweed consumption of available products on the diet quality of Australian consumers.

## Figures and Tables

**Table 1:** Demographic characteristics of the study participants (*n* = 1403).

Sample Characteristics	Frequency (*n*)	Percent (%)
Age		
19–24	653	46.5
25–30	750	53.5
Gender		
Female	1248	89.0
Male	73	5.2
Non-binary/other/prefer not to say	82	5.8
Highest education level		
Primary school	5	0.4
High school or equivalent	319	22.7
Diploma, certificate or equivalent	259	18.5
Tertiary level, equivalent, or above	810	57.7
Prefer not to say	10	0.7
Annual household income (AUD)		
Under $40,000	316	22.5
$40,000–$59,999	231	16.5
$60,000–$99,999	349	24.9
$100,000 or over	368	26.2
Prefer not to say	139	9.9
State of residence		
New South Wales	442	31.5
Victoria	360	25.7
Queensland	334	23.8
South Australia	92	6.6
Western Australia	82	5.8
Australian Capital Territory	54	3.8
Tasmania	34	2.4
Northern Territory	5	0.4
Geographical classification		
Metropolitan	1095	78.0
Rural	266	19.0
Metropolitan/rural	42	3.0

**Table 2:** Importance of various influences on decision making around the purchase of seaweed (*n* = 1403).

Influence *	Importance of Influence on Decision Making (Frequency, Percentage) **			
	Not Applicable	Not at All/Slightly Important	Moderately Important	Very Important
Taste	*n* = 6, 0.4%	*n* = 69, 4.9%	*n* = 178, 12.7%	*n* = 1150, 82.0%
Cost	*n* = 6, 0.4%	*n* = 206, 14.7%	*n* = 464, 33.1%	*n* = 727, 51.8%
Ease of preparation	*n* = 38, 2.7%	*n* = 283, 20.2%	*n* = 468, 33.4%	*n* = 614, 43.8%
Health	*n* = 11, 0.8%	*n* = 374, 26.7%	*n* = 436, 31.1%	*n* = 582, 41.5%
Freshness	*n* = 55, 3.9%	*n* = 396, 28.2%	*n* = 406, 28.9%	*n* = 546, 38.9%
Friend/family preference	*n* = 160, 11.4%	*n* = 840, 59.9%	*n* = 240, 17.1%	*n* = 163, 11.6%

* The influences are presented in descending order from most to least important. ** The four-point Likert scale is an amended version of the Likert scale used in the survey tool (see Methods 2.3).

**Table 3:** Frequency of seaweed consumption at different settings (*n* = 1403).

Setting *	Consumption Frequency (Frequency, Percentage) **					
	Never	Less Than Once Monthly	Monthly	Fortnightly	Weekly	Daily
Home prepared	*n* = 281, 20.0%	*n* = 447, 31.9%	*n* = 244, 17.4%	*n* = 165, 11.8%	*n* = 224, 16.0%	*n* = 42, 3.0%
Sushi bar/train	*n* = 60, 4.3%	*n* = 557, 39.7%	*n* = 427, 30.4%	*n* = 223, 15.9%	*n* = 129, 9.2%	*n* = 7, 0.5%
Restaurant	*n* = 151, 10.8%	*n* = 665, 47.4%	*n* = 371, 26.4%	*n* = 136, 9.7%	*n* = 77, 5.5%	*n* = 3, 0.2%
Fast food/takeout	*n* = 615, 43.8%	*n* = 431, 30.7%	*n* = 204, 14.5%	*n* = 93, 6.6%	*n* = 58, 4.1%	*n* = 2, 0.1%

* The settings are presented in descending order from the most to least frequently consumed. ** The six-point Likert scale presented is an amended version of the Likert scale used in the survey tool (see Methods 2.3).

**Table 4:** Motivators and barriers of seaweed consumption, as reported by participants.

Question (No.)	Theme	Frequency (*n*)	Percentage (%)	Representative Quotes *
Advantages (8)	Flavour profile	1248	89.1	“As a vegetarian, I find certain types of seaweed really bring out the umami flavour that is traditionally brought to the dish by non-vegetarian ingredients.” “Helps combat my cravings for salty, processed foods. The taste is unique and salty and satisfying.”
	Desirable nutrient content	688	49.1	“Contains many essential vitamins, nutrients and fibre with low calories.” “Good plant-based source of vitamins and minerals.” “It’s high in minerals such as iodine and magnesium, which are common deficiencies.”
	Health benefits	625	44.5	“Low calorie, savoury snack alternative.” “I feel that it is a great alternative to salt to sprinkle on dinner.” “It’s fantastic for your health.”
	Other sensory characteristics	274	19.6	“Aesthetically pleasing.” “Comes in a variety of textures—crunchy seaweed chips, soft seaweed wrapped sushi, fresh seaweed in soup.” “Feels nice in mouth.”
	Versatility	203	14.5	“It’s tasty and versatile to use in many dishes.” “Incorporating seaweed into my diet has pushed me to expand my diet and cooking skills.” “There are so many different ways you can eat it!”
	Convenience	162	11.6	“It is a delicious and convenient snack.” “They’re light, convenient and easy to carry.” “Easy to store, no fridge required and light in weight so (I) can carry it around all day.”
	Affordable price point	130	9.3	“Easy to incorporate into meals and inexpensive.” “Super cheap to buy proper seaweed in bulk.” “Cheap alternative, great for a little snack.”
	Sushi	122	8.7	“(Seaweed) makes eating sushi less messy.” “I LOVE to make sushi, so it is an essential in my kitchen cupboards.” “It holds delicious sushi fillings together.”
	Environmentally sustainable	106	7.6	“Supports biodiversity and sustainability.” “(I am) pretty sure seaweed farming can be used to combat climate change.” “Sustainable source of food.”
Disadvantages (9)	Undesirable side-effects	508	38.0	“Seaweed breath.” “Gets visibly stuck in your teeth.” “Very messy, (I) get flakes everywhere.”
	Unaffordable price point	428	32.0	“These nice seaweed snacks I like are super expensive gram for gram.” “It is expensive, like all health halo foods.” “More expensive than it should be.”
	Lack of accessibility	399	29.9	“(I) can’t grow it myself.” “Not readily available at Western supermarkets.” “Hard to find a range of varieties.”
	Other sensory characteristics	340	25.4	“Some brands have an overpowering smell and are oily.” “Fingers get oily.” “Smells like the ocean.”
	Undesirable flavour profile	262	19.6	“Can sometimes be overwhelmingly fishy in taste.” “Some varieties have a very strong flavour which can overtake a meal.” “It’s often seasoned with too much salt.”
	Undesirable packaging	241	18.0	“Packaging is often in languages other than English (language barrier).” “Most seaweed snack packs contain too few sheets of seaweed and it’s not satisfying enough, so I have to open multiple packs.” “Brands promote how good seaweed is for the environment, yet it mostly is still packaged in plastic.”
	Undesirable processing	239	17.9	“If you buy big packs it tends to go stale before you finish it.” “Often has a lot of oil in it, in commercial products.” “Hard to get good quality consistently.”
	Socially unacceptable	157	11.8	“Stigma—outside of Asian cuisines, not a lot of people have popularised seaweed as food.” “Lack of understanding/discrimination from others.” “I can’t convince my friends to try it.”
	Undesirable nutrient content	150	11.2	“If eaten in excess amounts and for long periods of time, can result in high levels of iodine.” “Most seaweed food items contain a lot of salt.” “Often comes with high sodium foods.”
	Inadequate knowledge, skills, and awareness	148	11.1	“I don’t know many ways to cook it other than sushi.” “Hard to incorporate into lots of meals.” “It isn’t used in a lot of Western cooking, so it’s difficult to find new recipes.”
	Unsatiating	115	8.6	“Low energy source – misconception that it’s a good snack on its own.” “Never feel completely satisfied after eating a pack.” “Not very filling, more of an addition than a full meal or snack.”
	Concern regarding environmental sustainability	114	8.5	“You can’t know if the seaweed was farmed sustainably.” “Very hard to find organic or locally farmed seaweed.” “Potentially contaminated with heavy metals.”
Enablers (10)	Greater accessibility	972	71.8	“If seaweed was more commonly used in cafes/restaurants.” “More products at convenience stores/petrol stations/general grocery chains.” “Better resources about foraging seaweed.”
	Affordable price point	621	45.9	“Available in major grocery stores at Asian supermarket prices.” “Cheaper prices so it would be less of a ‘treat’ and something that could be had more often.” “Cheaper options (e.g., sushi, onigiri) in convenience stores.”
	Desirable packaging	391	28.9	“Less plastic packaging, I want to satisfy my cravings without worrying about singlehandedly killing the planet.” “Clearer labelling in English (since many seaweed items are imported).” “If it came in vacuum sealed packs to buy in bulk.”
	Greater diversity of options	384	28.4	“Maybe if there were more supermarket products containing seaweed i.e., muesli bars or something. I would probably eat seaweed ice-cream not going to lie.” “More flavours of seaweed snacks.” “Sold in different forms (not just dried).”
	Opportunity for more targeted promotion	280	20.7	“More seaweed posts on social media regarding the benefits of seaweed, what brand to buy, etc.” “Better advertising so I remember it’s an option.” “Advertised more in media (including recipes).”
	Greater knowledge and skills	111	8.2	“More time and knowledge to prepare seaweed-containing foods.” “Knowing what to do with seaweed at home (other than sushi).” “Better cooking skills/knowledge.”
	Desirable processing	105	7.8	“If it didn’t spoil quickly.” “If seaweed crisps had olive oil and not oils like sunflower and canola.” “More consistent quality between brands.”
	Improved sensory characteristics	95	7.0	“Have it smell less like fish??” “Create more seaweed products that taste nice instead of just focusing on the health.” “Less chewy sometimes (i.e., Japanese seaweed salad).”
	Environmentally sustainable sourcing	79	5.8	“Clearer labelling/communication of where it is sourced from & how.” “More information available so I can be sure I am eating sustainably farmed seaweed.” “More Australian made and owned products.”
	Greater social acceptance	77	5.7	“If people know about it more, then I would be more comfortable sharing it at parties and what not.” “If seaweed was normalised as a food (most people see it as a foreign ingredient).” “More normalised in Western cooking.”
Barriers (11)	Lack of accessibility	741	59.5	“Living in a rural town it is expensive and rare to find.” “I don’t know how to forage for it and assume I’m not legally meant to.” “Not all forms of seaweed are available, may need to go to a specialty shop or Asian grocer.”
	Unaffordable price point	579	46.5	“The cost prevents me from eating it as often as I’d like.” “Good quality/organic seaweed is quite expensive.” “Sometimes seaweed snacks are not worth the price.”
	Undesirable packaging	237	19.0	“I don’t like how much plastic packaging it usually has so I try not to buy it too often.” “Packaging typically doesn’t display much nutritional information.” “Stockists almost never sell in bulk quantities.”
	Lack of product diversity	209	16.8	“I only have access to nori, dulse and wakame so I run out of dishes to use it in.” “Not common in shops except in crackers and sushi.” “If it’s in local stores, it’s expensive and really always the same product with no variety.”
	Undesirable processing	157	12.6	“Poor or untrustworthy quality.” “Shape (is) hard to use in cooking, e.g., long dried strands.” “Favourite crunchy snack seaweeds often cooked in unhealthy oils.”
	Lack of seaweed-related knowledge	174	12.4	“Not sure how to make it more versatile.” “Not wanting to spend (money) on stuff I don’t know how to use properly.” “Sushi is time consuming to make and I don’t know what else to do with seaweed.”
	Social unacceptance	153	12.3	“I rarely eat it in public due to the perception and judgement of others.” “Lack of normalisation in majority of Australian society.” “Family refuses to try it so I don’t bother buying it at times.”
	Undesirable flavour profile	132	10.6	“Occasionally the taste can be strong and off-putting.” “When (processors are) adding salt to already salty seaweed.” “Sometimes they are overly seasoned to mask the taste of the sea.”
	Undesirable sensory characteristics	112	9.0	“Sometimes I have textural issues with it when it is not crunchy.” “I only eat seaweed at home due to the overpowering smell of it which is not great in my workplace.” “Doesn’t appear appetising.”
	Inconvenient to prepare or purchase	104	8.3	“(I will) usually only eat it at a restaurant or take away.” “Sushi is time consuming to make and I don’t know what else to do with seaweed.” “Getting to Asian supermarkets to source the best types of seaweed snacks can be inconvenient.”
	Undesirable side-effects of consumption	97	7.8	“Strong seaweed breath.” “Can be a bit greasy and messy – so not much of an on-the-go snack.” “I have to make sure it’s not stuck on my teeth or face if I’m eating it around other people.”

* The table presents the themes that correlated to ≥5.0% of respondents. The themes are presented in descending order from most to least frequently reported.

## Data Availability

Data is contained within the article or Supplementary material.

## Supplementary Materials

The following supporting information can be downloaded at: https://www.mdpi.com/article/10.3390/foods11193052/s1, Table S1: Survey questions and responses.
