# Supplementary material for: Factors Influencing the Consumption of Seaweed amongst Young Adults
Source: Foods. 2022 Oct 1;11(19):3052. doi: 10.3390/foods11193052 (PMC9563983; doi:10.3390/foods11193052)
Supplement: Supplementary file 1 [file foods-11-03052-s001.zip › foods-1924861-supplementary.pdf]

foods MDPI

Supplementary Materials

Survey Questions

Table S1: Survey questions and responses.*

Survey Question	Response Type/Options
1. How old are you (in years)?	• < 19<br>• 19-24<br>• 25-30<br>• > 30
2. Have you eaten seaweed on at least one occasion over the past 12 months? For example, sushi wrapped in seaweed, seaweed crackers or miso soup.	• Yes<br>• No<br>• Unsure
3. Are you comfortable completing this survey in English?	• Yes<br>• No
4. What is your gender?	• Male<br>• Female<br>• Non-binary/Other<br>• Prefer not to say
5. What is your highest level of education?	• Completed primary school<br>• Completed high school or equivalent<br>• Completed diploma, certificate or equivalent<br>• Completed tertiary level education, equivalent, or above<br>• Prefer not to say
6. What is your average household income? (in $AUD)	• Under $40,000<br>• $40,000-$59,999<br>• $60,000-$99,999<br>• $100,000 or over<br>• Prefer not to say
7. What is your postcode?	[Free text]
8. Please list three of the greatest **advantages** of eating seaweed (what are the 3 best things about eating seaweed).	[Free text]<br>[Free text]<br>[Free text]
9. Please list three of the greatest **disadvantages** of eating seaweed (what are the 3 worst things about eating seaweed).	[Free text]<br>[Free text]<br>[Free text]

10. Please list three factors that would **enable (make it easier)** for you to eat seaweed.
    [Free text]
    [Free text]
    [Free text]

11. Please list three factors that make it **difficult (harder)** for you to eat seaweed.
    [Free text]
    [Free text]
    [Free text]

12. How important are the following factors in influencing your decision to purchase seaweed? Please respond to all options.

	Not applicable	Not at all important	Slightly important	Moderately important	Very important	Extremely important
Taste	○	○	○	○	○	○
Cost	○	○	○	○	○	○
Freshness	○	○	○	○	○	○
Ease of preparation	○	○	○	○	○	○
Friend/Family preference	○	○	○	○	○	○
Health	○	○	○	○	○	○

13. Which sources of seaweed information would you use? Select all that apply.
    - Books
    - Brochures/Handouts
    - Consumer advocacy groups
    - Environmental groups
    - Family/Friends
    - General practitioners
    - Government publications
    - Health newsletter
    - Internet
    - Media
    - Nutritionists/Dietitians
    - Point of purchase

14. What would be the best way (your preferred way) for you to get information about seaweed? Select one.
    - Books
    - Brochure/Handouts
    - Environmental groups
    - Family/Friends
    - General practitioners
    - Health newsletter
    - In-store signs
    - Internet
    - Media
    - Nutritionists/Dietitians
    - Point of purchase

15. Can you recall any varieties or types of seaweed?
    - Yes (please specify): [Free text]
    - No

16. [Display this question if answer to Q.15 is yes] What varieties or types of seaweed do you like to eat?
    [Free text]

17. When do you eat seaweed? Select all that apply.
    - Lunch
    - Dinner
    - Snacks
    - Special occasion (please specify): [Free text]
    - Other (please specify): [Free text]

18. How often do you eat seaweed from the following locations? Please respond to all options.

	Never	Every few months	Less than once per month	Monthly	Fortnightly	Weekly	Daily	More than once daily
Home prepared	○	○	○	○	○	○	○	○
Resturant	○	○	○	○	○	○	○	○
Sushi bar/sushi train	○	○	○	○	○	○	○	○
Fast food or takeout (please specify)	○	○	○	○	○	○	○	○
Takeout (if different to the first) (please specify)	○	○	○	○	○	○	○	○

19. Please rate your level of agreement with each of the following statements on a scale from very strongly disagree to very strongly agree.

	Very strongly disagree	Strongly disagree	Disagree	Neither agree nor disagree	Agree	Strongly agree	Very strongly agree
I am constantly sampling and trying new and different foods.	○	○	○	○	○	○	○
I don't trust new foods.	○	○	○	○	○	○	○

	If I don't know what is in a food, I won't try it.	○	○	○	○	○	○	○
	I like foods from different countries.	○	○	○	○	○	○	○
	Ethnic food looks too weird to eat.	○	○	○	○	○	○	○
	At dinner parties, I will try a new food.	○	○	○	○	○	○	○
	I am afraid to eat things I have never had before.	○	○	○	○	○	○	○
	I am very particular about the foods I will eat.	○	○	○	○	○	○	○
	I will eat almost anything.	○	○	○	○	○	○	○
	I like to try new ethnic resturants.	○	○	○	○	○	○	○

*The survey includes an adapted version of the tool used by Hicks et al to assess consumer beliefs and knowledge regarding seafood consumption (Q.13, Q.14, Q.18) [31], and the validated Food Neophobia Scale by Pliner and Hobden (Q.19) [25].
